# DGHNN: a deep graph and hypergraph neural network for pan-cancer related gene prediction

**DOI:** 10.1093/bioinformatics/btaf379

**Published:** 2025-06-28

**Authors:** Bing Li, Xin Xiao, Chao Zhang, Ming Xiao, Le Zhang

**Affiliations:** College of Computer Science, Sichuan University, Chengdu, 610000, China; Department of Thoracic Surgery, West China Hospital of Sichuan University, Chengdu, 610000, China; West China Biomedical Big Data Center, West China Hospital, Sichuan University, Chengdu, 610000, China; College of Computer Science, Sichuan University, Chengdu, 610000, China; College of Computer Science, Sichuan University, Chengdu, 610000, China

## Abstract

**Motivation:**

Studies on pan-cancer related genes play important roles in cancer research and precision therapy. With the richness of research data and the development of neural networks, several successful methods that take advantage of multiomics data, protein interaction networks, and graph neural networks to predict cancer genes have emerged. However, these methods also have several problems, such as ignoring potentially useful biological data and providing limited representations of higher-order information.

**Results:**

In this work, we propose a pan-cancer related gene predictive model, the DGHNN, which takes biological pathways into consideration, applies a deep graph and hypergraph neural network to encode the higher-order information in the protein interaction network and biological pathway, introduces skip residual connections into the deep graph and hypergraph neural network to avoid problems with training the deep neural network, and finally uses a feature tokenizer and transformer for classification. The experimental results show that the DGHNN outperforms other methods and achieves state-of-the-art model performance for pan-cancer related gene prediction.

**Availability and implementation:**

The DGHNN is available at https://github.com/skytea/DGHNN.

## 1 Introduction

Cancer-related study is very important in the field of biomedical research. Since the occurrence and progression of cancer are triggered by the accumulation of mutations in multiple cancer driver genes (Vogelstein *et al.* 2013), understanding the genetic basis of cancer occurrence and identifying pan-cancer related genes are the key goals of cancer-related research (Lawrence *et al.* 2014). In addition, the identification of pan-cancer related genes plays a crucial role in the precise therapy and personalized medical care of cancer patients (Kandoth *et al.* 2013, Vogelstein *et al.* 2013, Lawrence *et al.* 2014).

In recent years, several research projects, such as The Cancer Genome Atlas (TCGA) (Tomczak *et al.* 2015) and the International Cancer Genome Consortium (ICGC) (Hudson *et al.* 2010), have collected large amounts of multiomics data for both cancer and normal tissues, which offer rich resources for research on cancer mechanisms, identification of pathogenic genes, and development of personalized treatment strategies (Bailey *et al.* 2018, Priestley *et al.* 2019). For example, by analysing multiple types of omics data, researchers have identified the role of driver gene mutations and revealed the molecular heterogeneity among different cancer patients (Martincorena and Campbell 2015, Zhang and Zhang 2017, Zhang *et al.* 2018, Zhang *et al.* 2021a,b,c,d, You *et al.* 2022, Zhang *et al.* 2023a,b,c, Li *et al.* 2025). However, owing to the diversity of data sources (such as the genome, transcriptome and epigenome) and the complexity of data structures, designing effective algorithms and models for integrating heterogeneous data, deeply analysing potential information, and accurately predicting pan-cancer related genes have become major challenges in current research (Bashashati *et al.* 2013, Tomczak *et al.* 2015). In this study, pan-cancer related genes refer to those cancer genes which are collected by experts, high-confidence cancer genes which are mined from PubMed abstracts, and genes with altered expression and promoter methylation in at least one cancer type (Schulte-Sasse *et al.* 2021). Therefore, constructing methods and models that can fuse various data types to predict pan-cancer related genes is an important area of cancer research.

In previous studies, Schulte-Sasse *et al.* proposed the EMOGI method, which combines multiomics data and protein interaction network (PPI) information, and used a graph convolutional neural network (GCN) to construct a cancer gene predictive model (Schulte-Sasse *et al.* 2021). Specifically, EMOGI uses PPI networks to construct a graph in which genes are represented as nodes and multiomics data (gene mutation information, copy number variation, DNA methylation, gene expression, etc.) are represented as attributes of nodes. By integrating different types of biological data, EMOGI can capture more comprehensive signature information for cancer gene prediction. On the basis of EMOGI, the EMGNN method (Chatzianastasis *et al.* 2023) attempts to integrate the results of six different PPI networks to optimize cancer gene prediction, and the EMGNN prediction is interpreted through the integrated gradients at the model level and the gene pathways at the molecular level.

Despite the success of the above studies, several shortcomings remain. A major limitation is that only the protein interaction networks (PPIs) are well considered, whereas the gene interrelationships between biological pathways are not adequately considered. In biological processes, genes and proteins in the same biological pathway usually have close interactions since previous reports have shown that cancer-driving mutations can unlock the carcinogenic properties of cells by altering the activity of key pathways (Martínez-Jiménez *et al.* 2020). In addition, pathway and network analyses usually play important roles in cancer gene prediction, especially in predicting potential cancer genes on the basis of the locations of infrequently mutated genes in the pathways and their physical or regulatory interactions with commonly mutated genes (Reyna *et al.* 2020). Biological pathways also have been used and combined by recent study for cancer recurrence prediction and biomarker discovery, DeepKEGG, proving the effectiveness of pathways information (Lan *et al.* 2024). However, owing to the complexity and diversity of biological pathway data, normal graph models have difficulty effectively representing and using this information. Therefore, how to design an effective cancer gene predictive model that encodes and combines biological pathways, becomes the first scientific problem in the study.

In addition, model construction for data mining is important. Although shallow graph neural networks are widely used in many tasks, they are unable to capture information of higher-order neighbour nodes and are insufficient for capturing global information for complex graph structures or data that consisting of many nodes, thus resulting in limited expression ability for large and complex graphs and potentially degrading the performance of the model (Xu *et al.* 2019, Chen *et al.* 2020). Deep graph neural networks (Chen *et al.* 2024) can better combine global information and capture complex relationships among higher-order neighbour nodes through multilayer structures to obtain higher-order information in the graph, but their applications still encounter many challenges, including vanishing gradients, exploding gradients, and excessive smoothing in the training process of deep networks, thus increasing the difficulty of model training and optimization (Kipf and Welling 2017, Oono and Suzuki 2020, Gao *et al.* 2021, Song *et al.* 2022, Gao *et al.* 2023). Therefore, how to develop such a deep graph neural network that cannot only obtain higher-order information from graphs but also solve the problems of vanishing gradients and exploding gradients, which may occur in the training process of deep graph neural networks, becomes the second scientific problem in this study.

After data mining and feature embedding, building an effective classifier to obtain prediction results is crucial. In recent years, with the rapid development of the transformer architecture and large language models (LLMs) (Jahan *et al.* 2024, Jiang *et al.* 2024, Gao *et al.* 2025, You *et al.* 2025), several studies have attempted to use LLMs for a variety of tasks, including classification tasks. In the field of text classification, LLMs have made good progress (Dinh *et al.* 2022, Hegselmann *et al.* 2023, Sun *et al.* 2023), and LLMs have also become indispensable in biomedical data analysis research (Lan *et al.* 2025). However, for numerical and tabular data, LLMs often fail to fully understand and process features because numerical and tabular data are simply numbers or categories rather than structured semantic and contextual information such as text. Therefore, how to build up a feature tokenizer for the numerical data and the efficiently use of the embeddings to obtain accurate prediction results becomes the third scientific problem in the study.

In response to the above three scientific problems, this study proposes three innovations. First, we developed a hypergraph model (Bai *et al.* 2021) based biological pathway modelling method, which can efficiently encode biological pathway information and more comprehensively capture the complex relationships between genes and pathways. Second, we developed a deep graph neural network model based on skip residual connections, which can extract more higher-order information from large graphs, overcome the limitations of shallow networks, effectively solve the problem of over smoothing in deep graph neural networks, and increase the performance of pan-cancer related gene prediction. Finally, inspired by the FT transformer (Gorishniy *et al.* 2021), we propose a predictive classifier based on feature tokenizer and transformer, which not only makes full use of feature embeddings for accurate prediction but also has good compatibility and can maintain the robustness and flexibility of the model when new features are introduced.

In conclusion, this study proposes a pan-cancer related gene predictive model, the DGHNN, which uses the properties of graphs and hypergraphs to integrate a PPI network (Zhao *et al.* 2020, Zhang *et al.* 2024a,b,c,d), biological pathway information (Zhang *et al.* 2016, Xia *et al.* 2017, Zhang *et al.* 2019, Liu *et al.* 2020, Lai *et al.* 2022), and multiomics data. A deep neural network based on skip residual connections is constructed to extract more comprehensive higher-order information from large graphs, and the predictions are made based on feature tokenizer and transformer structure. As a result, DGHNN outperforms other previous studies on all 6 well-known public datasets, which demonstrates that our model has better prediction ability.

## 2 Methods

### 2.1 Overview of the proposed pan-cancer related gene predictive model (DGHNN)

**Figure 1.**
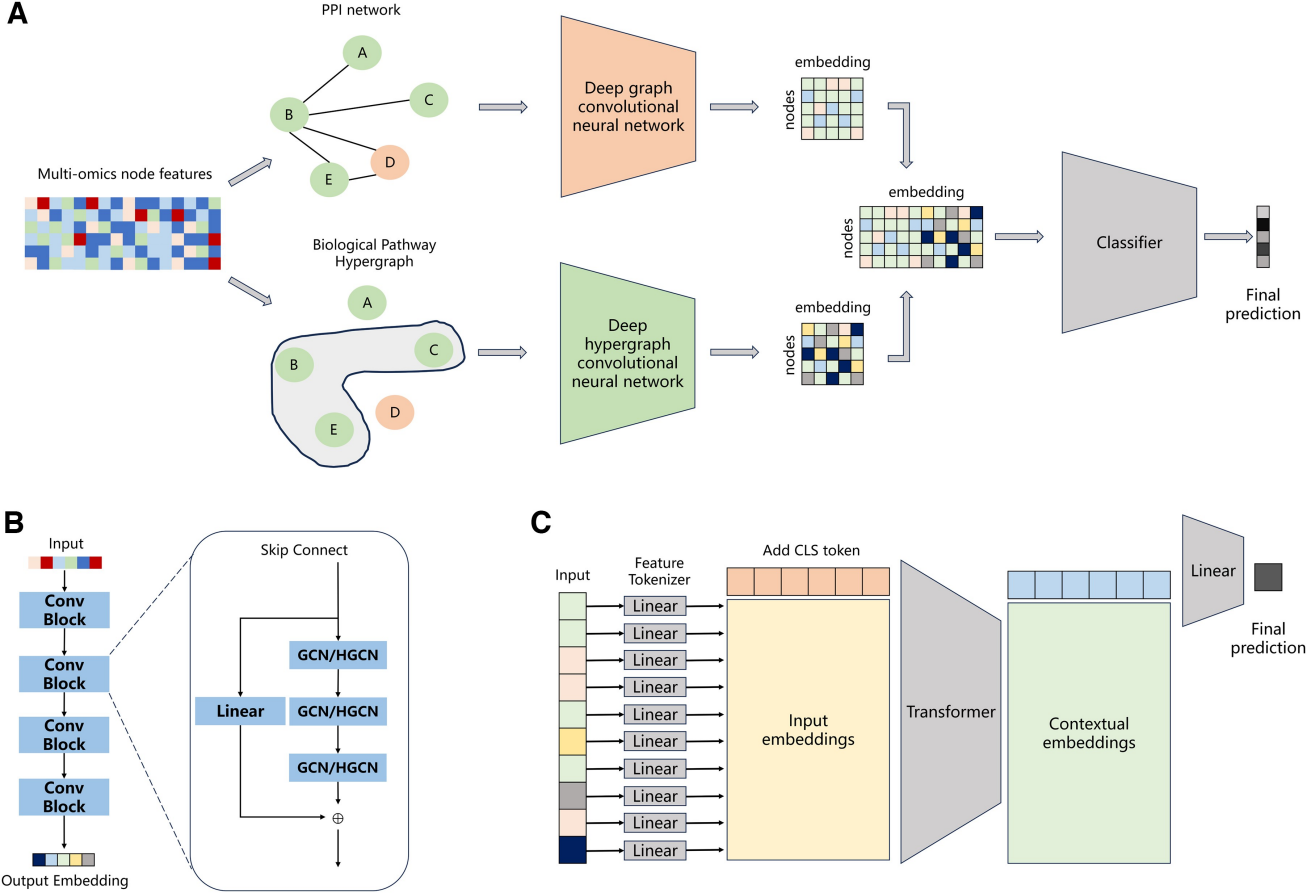
Overview of the DGHNN. (A) Structure of the DGHNN, (B) deep graph/hypergraph neural network model based on skip residual connections, and (C) classification module based on feature tokenizer and transformer.

This study proposes a pan-cancer related gene prediction model, the DGHNN. Figure 1A shows the structure of the DGHNN.

The input data are {G, HG, V}, where G represents the protein interaction network data, HG represents the biological pathway hypergraph network data, and V represents the nodes in the two graphs and the corresponding attribute values of the nodes, which are the multiomics data of each gene node.

Our pan-cancer related gene predictive model first aggregates multiomics network data and nodes by a graph convolutional neural network and a hypergraph convolutional neural network, respectively, and then extracts feature embeddings based on the corresponding graph and hypergraph.


(1) Egraph=DeepGCN(G, V)

(2) Ehypergraph=DeepHGCN(HG, V)

DeepGCN is composed of a series of graph convolution modules. Each graph convolution module (Fig. 1B) consists of a three-layer graph convolution neural network and skip connections. DeepHGCN is composed of a series of hypergraph convolution modules, which are like the graph convolution modules with a three-layer hypergraph convolutional neural network and skip connections. $E_{\mathrm{graph}}$ represents the feature embeddings extracted from the graph convolutional neural network, and $E_{\mathrm{hypergraph}}$ represents the feature embeddings extracted from the hypergraph convolutional neural network.

After embedding the features of the graph and hypergraph, we fuse their feature information. In this study, we concatenate the features from the graph and hypergraph as shown in Equation (3).

(3) Emerge=Concat(Egraph,Ehypergraph)

Finally, we input the merged feature embeddings $E_{\mathrm{merge}}$ into the classification module (Fig. 1C) to predict whether the gene nodes correspond to pan-cancer related genes by Equation (4).

(4) Pred=CLS(Emerge)

There are three technical components of the DGHNN: a graph and hypergraph neural network, a deep graph and hypergraph neural network based on skip residual connections and a classification module based on feature tokenizer and transformer. Owing to length limitations for the manuscript, additional details about these three parts can be found in the Supplementary Material, available as supplementary data at *Bioinformatics* online.

### 2.2 Datasets and experimental setup

To ensure a fair comparison with the previous methods (Schulte-Sasse *et al.* 2021, Chatzianastasis *et al.* 2023), we used their corresponding datasets with the same data splits. Additional details about the datasets can be found in the Supplementary Material, available as supplementary data at *Bioinformatics* online.

Moreover, the following are the hyperparameters used in the experiment and our setup: the number of nodes of the hidden layer is set to 64, the learning rate was set to 1e-4, the decay rate was set to 1e-5, the dropout rate was set to 0.1, and the number of training epochs was set to 3000. For the classifier, the number of embedded nodes was set to 16, the number of layers was set to 4, the number of transformer heads was set to 4, and the dropout rate of attention and forward processing was set to 0.1. In addition, we used BCELoss as the loss function and ADAM for training (Kingma and Ba 2015).

## 3 Results

### 3.1 Setup for model performance and comparison

To answer the three scientific questions mentioned above and further explore and prove the effectiveness of the deep graph and hypergraph neural network proposed in this study, we conducted several experiments and comparisons, as discussed below. And AUROC and AUPRC were used to evaluate the performance of the model (Richardson *et al.* 2024).

**Figure 2.**
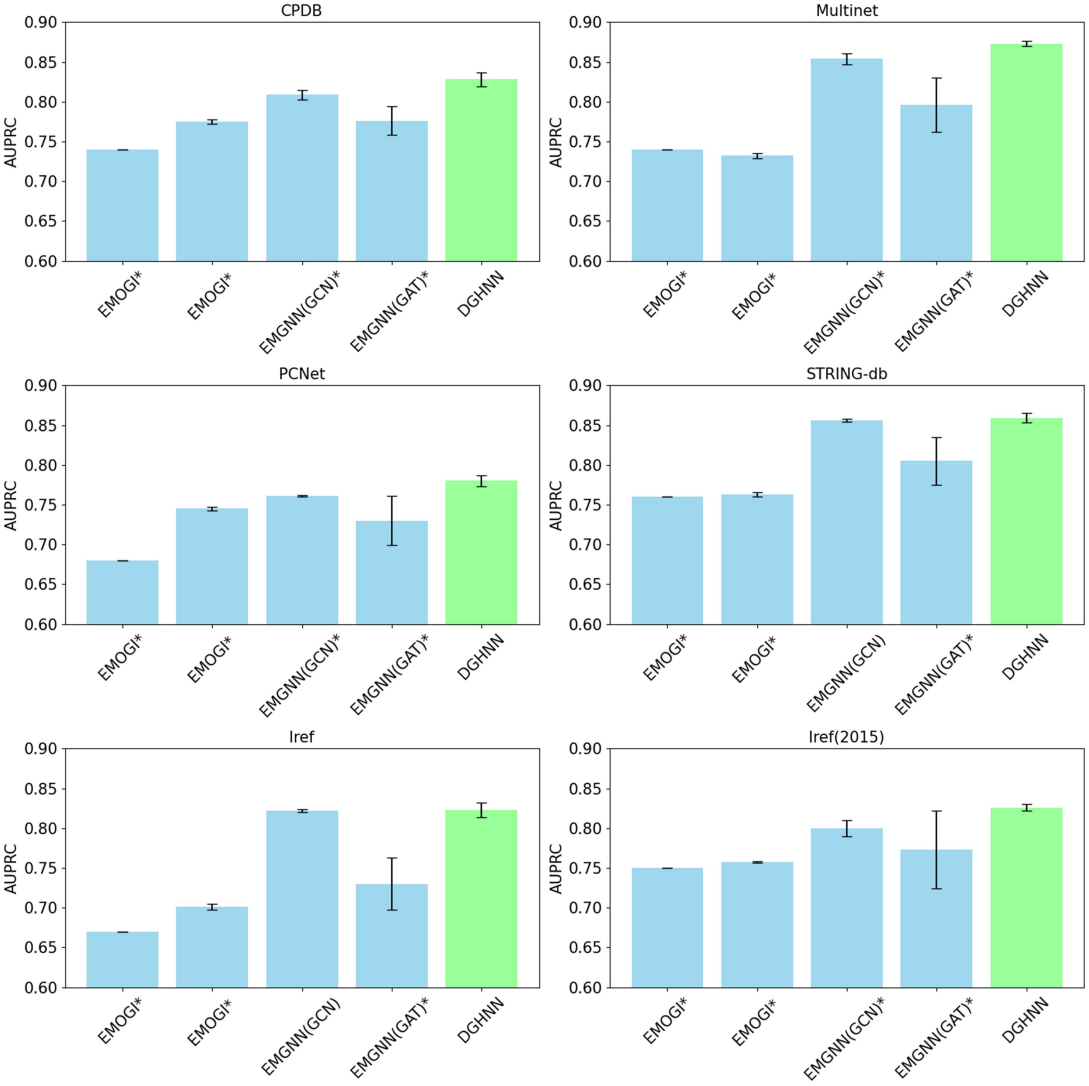
AUPRCs of different models on all six datasets. The * symbol indicates that the results are significantly different.

Table 1 and Fig. 2 compare the performance of the DGHNN with those of previous commonly used models, namely, EMOGI (Schulte-Sasse *et al.* 2021, Hong *et al.* 2022), EMGNN(GCN) (Chatzianastasis *et al.* 2023), and EMGNN(GAT) (Chatzianastasis *et al.* 2023), on six datasets, namely, CPDB, Multinet, PCNet, STRING-db, Iref, and Iref(2015).

**Table 1.** AUPRC results of five models on all six datasets.^a^

Method	CPDB	Multinet	PCNet	STRING-db	Iref	Iref (2015)
EMOGI (Schulte-Sasse *et al.* 2021)	0.74*	0.74*	0.68*	0.76*	0.67*	0.75*
EMOGI (Hong *et al.* 2022)	0.775 ± 0.003*	0.732 ± 0.003*	0.745 ± 0.002*	0.763 ± 0.003*	0.701 ± 0.004*	0.757 ± 0.001*
EMGNN(GCN) (Chatzianastasis *et al.* 2023)	0.809 ± 0.006*	0.854 ± 0.007*	0.761 ± 0.001*	0.856 ± 0.002	0.822 ± 0.002	0.800 ± 0.010*
EMGNN(GAT) (Chatzianastasis *et al.* 2023)	0.776 ± 0.018*	0.796 ± 0.034*	0.730 ± 0.031*	0.805 ± 0.030*	0.730 ± 0.033*	0.773 ± 0.049*
DGHNN	**0.828 ± 0.009**	**0.873 ± 0.003**	**0.780 ± 0.007**	**0.859 ± 0.006**	**0.823 ± 0.009**	**0.826 ± 0.004**

a The result with

* represents that the result of this method is statistically different from the one of DGHNN on this dataset, which is calculated by *T* test and the *P*-value threshold is set to .05. The bold values represent the best performances in the table.

In addition, we conducted ablation experiments with the following models: (i) DGHNN_L: a linear layer is used as the final classifier; (ii) DGHNN_NO_H: the biological pathway information based on the hypergraph neural network is deleted, and only the part of the graph neural network is retained; (iii) DGHNN_NO_G: the protein–protein interaction information based on the graph neural network is deleted, and only the part of the hypergraph neural network is retained; and (iv) SGHNN: a shallow neural network model with only three layers. The performance of these models is shown in Tables 1 and Table 2, available as supplementary data at *Bioinformatics* online.

To ensure the reliability of the experimental data, all the experiments were run under five different random seeds, and the means and standard deviations of the results were recorded.

### 3.2 Effectiveness of the hypergraph neural network

To answer the first scientific question, how to design an effective cancer gene predictive model that encodes and combines biological pathways? We compared our proposed DGHNN model with the EMGNN(GCN) and DGHNN_NO_H models, which do not consider biological pathways, as shown in Table 1, Fig. 2, and Tables 1 and 2, available as supplementary data at *Bioinformatics* online.

According to Table 1 and Fig. 2, the DGHNN outperformed the previous state-of-the-art models on all six datasets (Chatzianastasis *et al.* 2023). The performance in terms of the area under the precision–recall curve (AUPRC) increased greatly on the CPDB, Multinet, PCNet, and Iref (2015) datasets and improved slightly on the other two datasets (STRING-db and Iref). The greatest increases occurred on Iref (2015) and Multinet, namely, from 0.8 and 0.854 to 0.826 and 0.873, respectively.

According to Tables 1 and 2, available as supplementary data at *Bioinformatics* online, the performance of the DGHNN increased statistically significantly on five datasets compared to that of the DGHNN_NO_H, and the difference between the performances of the DGHNN and DGHNN_NO_H on the remaining one dataset was nonsignificant by T-test (Zhang *et al.*  2021a,b,c,d, Zhang *et al.*  2023a,b,c, Huang *et al.* 2024, Ou *et al.* 2024, Xiao *et al.*  2024a,b,c, You *et al.* 2024), which demonstrates the effectiveness of using biological pathways and hypergraph convolutional neural networks.

These experimental data show that our DGHNN model outperformed the models in previous studies (Schulte-Sasse *et al.* 2021, Chatzianastasis *et al.* 2023) and achieved state-of-the-art performance for cancer gene prediction, which not only demonstrates the improved performance of the model after encoding and integrating biological pathway data but also proves the effectiveness of using hypergraph convolutional neural networks to develop the DGHNN.

### 3.3 Effectiveness of deep graph neural networks

To answer the second scientific question, how to develop such a deep graph neural network that cannot only obtain higher-order information from graphs but also solve the problems of vanishing gradients and exploding gradients, which may occur in the training process of deep graph neural networks. Our first task was to explore how many layers should be selected to achieve the best predictive performance for deep graph and hypergraph convolutional neural networks and to reach a balance between model performance and resource consumption.

**Figure 3.**
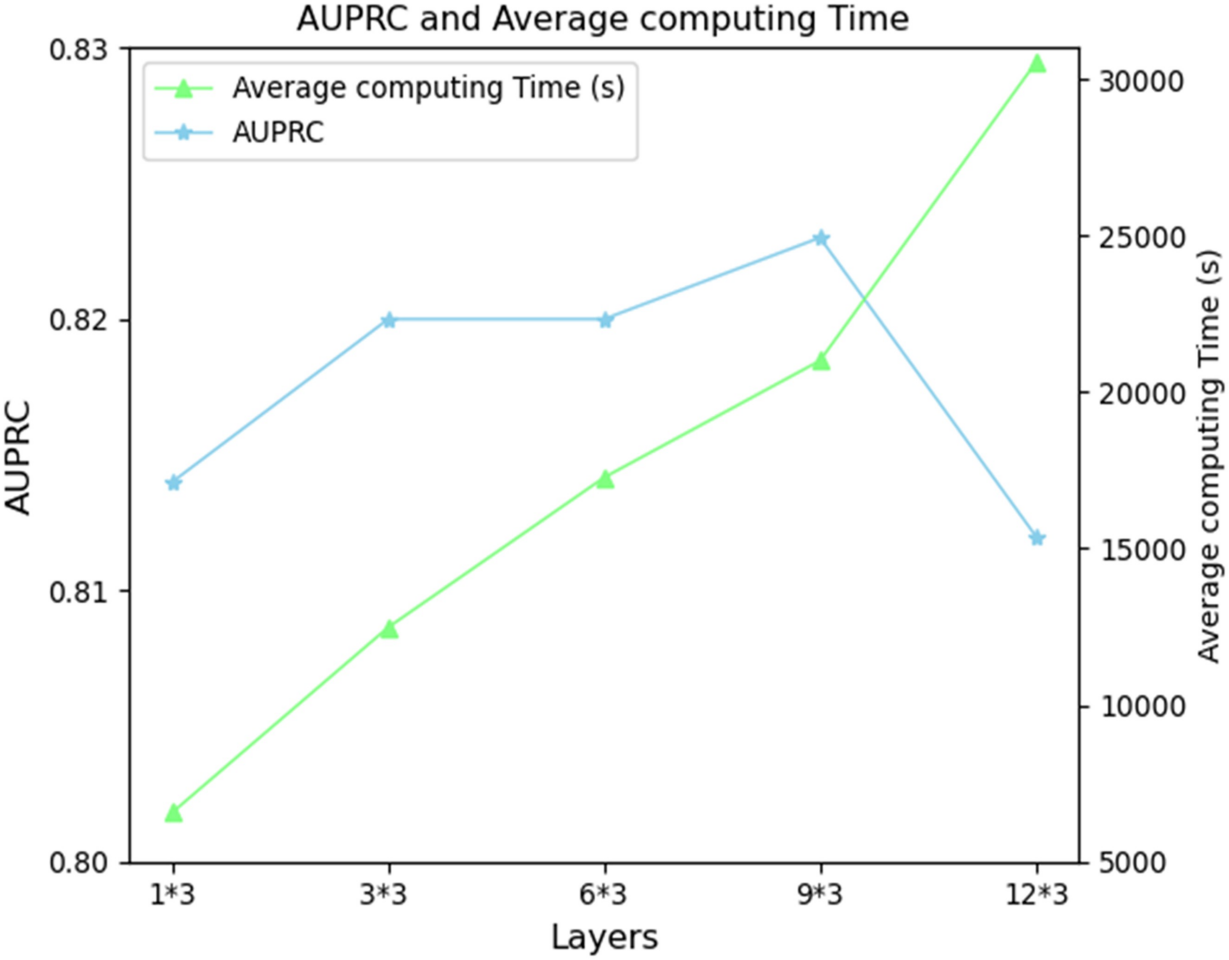
AUPRC results and time costs of different layers.

We chose the Iref dataset as an example to explore the possible numbers of layers. Since our deep graph neural network is based on a module of graph convolutional modules, each of which has a three-layer graph convolutional neural network, we tested the neural network with 1*3, 3*3, 6*3, 9*3, and 12*3 convolutional layers, and the final AUPRC performance and time costs are shown in Table 3, available as supplementary data at *Bioinformatics* online and Fig. 3.

Table 3, available as supplementary data at *Bioinformatics* online and Fig. 3 show that the performance of the DGHNN increased with increasing number of model layers. However, the performance of the DGHNN decreased when the number of model layers became very large, which implies that a very deep graph convolutional neural network decreases the model performance, although deep graph convolutional neural networks are good at obtaining higher-order information for complex graph structures. In addition, the average computing time cost for each experiment indicates that with an increase in the number of model layers, we need significant time and resources for computing, which greatly increases the resources requested for model training.

Considering the above model performance results and the average computation time, for the DGHNN model proposed in this study, a neural network with 9*3 convolutional modules was ultimately selected, which includes 27-layer graph convolutional neural networks with 9 skip residual connections, to guarantee the optimal model performance under an acceptable consumption of computing resources.

After that, we compared the DGHNN and the SGHNN (a shallow neural network model with only three layers) via ablation experiments to demonstrate the effectiveness of the deep graph neural network.

**Figure 4.**
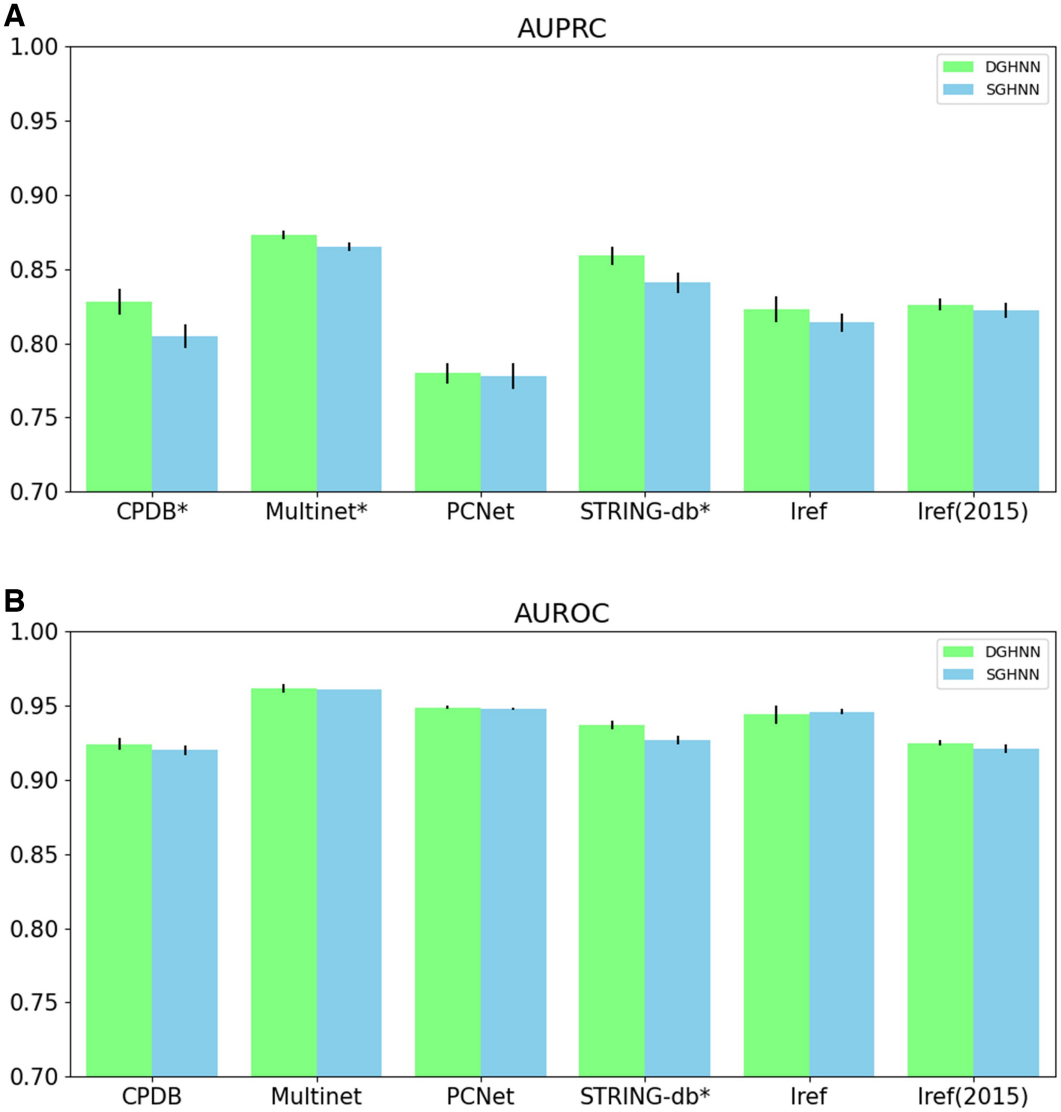
Comparisons of AUPRC and AUROC between the DGHNN and SGHNN models: (A) AUPRC and (B) AUROC results. The * symbol indicates that the results are significantly different.

According to the comparison results for AUPRC (Table 1, available as supplementary data at *Bioinformatics* online and Fig. 4A), DGHNN statistically significantly outperformed the SGHNN model on the CPDB, Multinet and STRING-db datasets, and the differences between the performances of the DGHNN and SGHNN on the remaining three datasets were nonsignificant. The comparison of the AUROC results (Table 2, available as supplementary data at *Bioinformatics* online and Fig. 4B) demonstrates that the DGHNN performed statistically significantly better than the SGHNN model on STRING-db, and the differences between the performance of the DGHNN and SGHNN on the remaining five datasets were nonsignificant.

**Figure 5.**
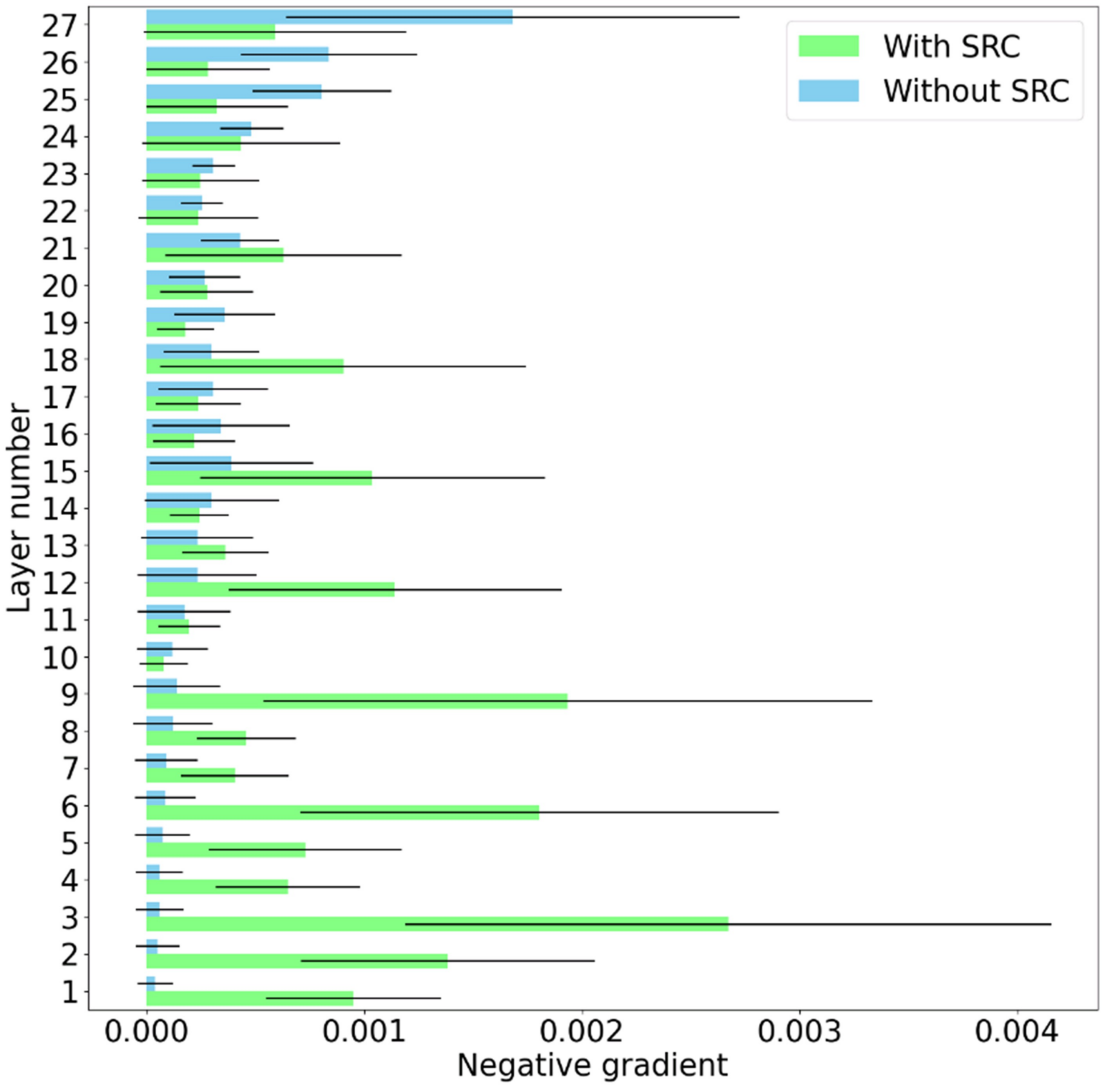
Negative gradient for the model with and without the skip residual connections. SRC is an abbreviation for skip residual connections.

To prove the effectiveness of skip residual connections in solving the problems of gradient vanishing and explosion, which may occur in the training of deep graph neural networks, Fig. 5 compares the gradients of the model with and without skip residual connections.

Here, we list the gradients of the 27 layers during model training (the first 100 epochs) and calculate the mean and standard deviation of the gradients to show the differences. Figure 5 shows that the model without skip residual connections had trainable gradients in the first several layers, but the gradient began to decrease rapidly as the number of layers increased. The vanishing gradient problem occurred in layers 1–10, which caused the weight update in layers 1–10 to become very slow or even almost stagnant, thus preventing the network from learning effectively from the input data.

In contrast, analysing the gradients of models with skip residual connections shows that the gradients increased after every three layers precisely because of the skip residual connections used in every three layers, which made the gradients of almost all layers trainable. Thus, Fig. 5 proves the effectiveness of skip residual connections.

Therefore, we not only conclude that deep graph neural networks can more effectively obtain higher-order information from graphs and achieve better performance but also prove that our proposed skip residual connections can effectively alleviate the vanishing gradient problem that may occur in the training of deep graph neural networks.

### 3.4 Effectiveness of the feature tokenizer and transformer for classification

**Figure 6.**
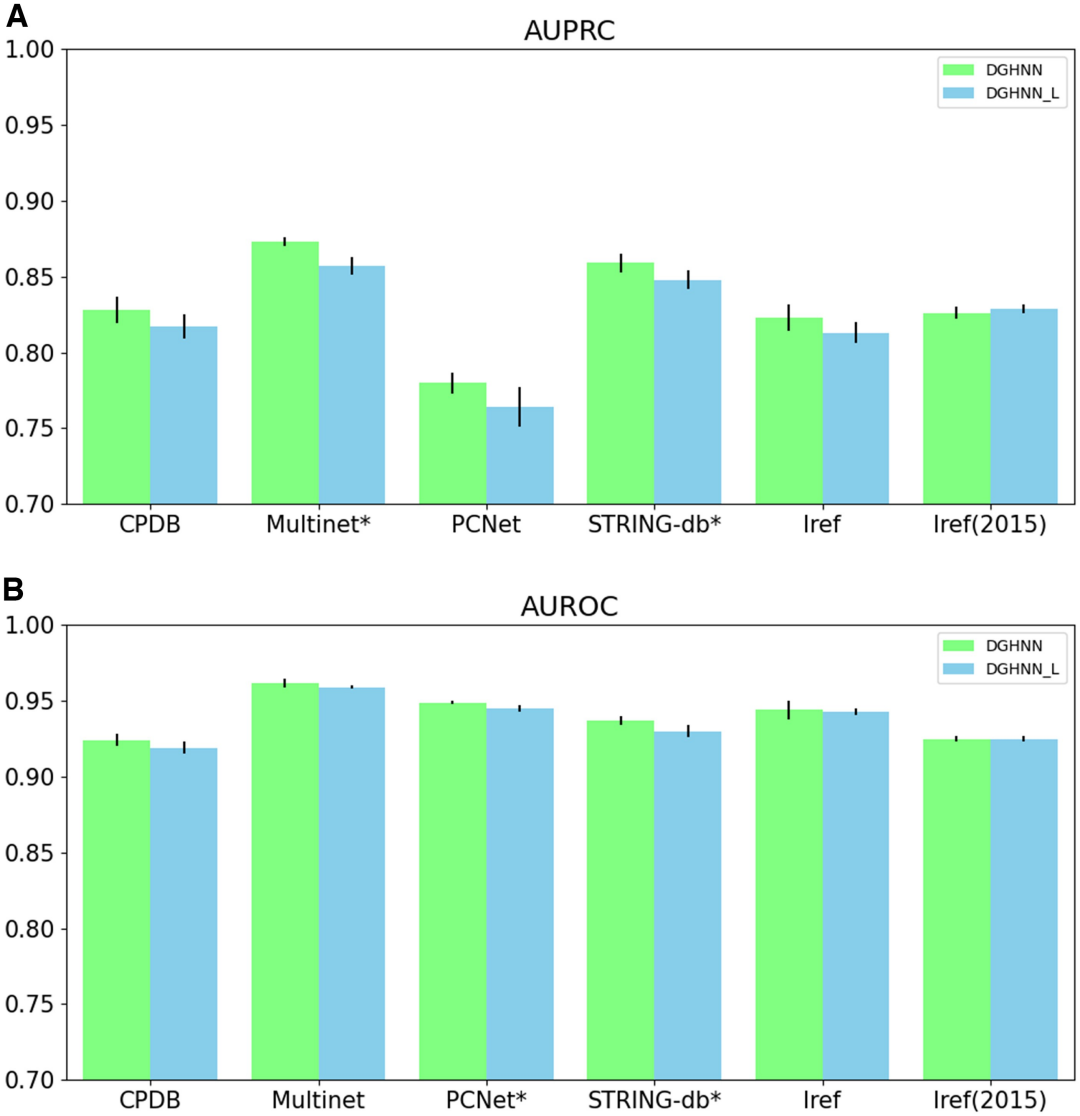
Comparisons of AUPRC and AUROC between the DGHNN and DGHNN_L model: (A) AUPRC and (B) AUROC results. The * symbol indicates that the results are significantly different.

To answer the third scientific question, how to build up a feature tokenizer for the numerical data and the efficiently use of the embeddings to obtain accurate prediction results. We compared our proposed DGHNN model with DGHNN_L, which uses a linear layer as the final classifier, as shown in Fig. 6 and in Tables 1 and 2, available as supplementary data at *Bioinformatics* online.

The comparison results in terms of AUPRC (Table 1, available as supplementary data at *Bioinformatics* online and Fig. 6A) show that the DGHNN performed statistically significantly better than the DGHNN_L model on Multinet and STRING-db, and the differences between the performances of DGHNN and DGHNN_L on the remaining four datasets were nonsignificant. The comparison of the AUROC results (Table 2, available as supplementary data at *Bioinformatics* online and Fig. 6B) shows that the DGHNN performed statistically significantly better than the DGHNN_L model on PCNet and STRING-db, and the differences between the performances of DGHNN and DGHNN_L on the remaining four datasets were nonsignificant.

According to the comparison results of AUPRC and AUROC, DGHNN outperformed DGHNN_L, which proves that our proposed classifier, which uses a feature tokenizer and transformer structure, can achieve better predictive performance.

## 4 Discussion and conclusions

This study developed a pan-cancer related gene predictive model based on deep graph and hypergraph neural networks by answering the three scientific questions mentioned above. The corresponding innovations are as follows: (i) we developed a hypergraph model-based biological pathway modelling method that can efficiently encode biological pathway information and more comprehensively capture the complex relationships between genes and pathways; (ii) we developed a deep graph neural network model based on skip residual connections and can extract more higher-order information from large graphs, overcome the limitations of shallow networks, and effectively solve the problem of over smoothing in deep graph neural networks to increase the performance of cancer gene prediction; and (iii) we proposed a predictive classifier based on feature tokenizer and transformer which not only makes full use of feature embeddings for accurate prediction, but also has good compatibility and can maintain the robustness and flexibility of the model when new features are introduced.

To demonstrate the validity of our proposed model, we conducted comprehensive experiments with both previous best-performing models (Chatzianastasis *et al.* 2023) and models for ablation experiments. All the experiments were conducted under five different random seeds, and the means and variances of the five results were used for statistical analysis. AUPRC and AUROC, which are listed in Table 1 and Supplementary Tables 1 and 2, available as supplementary data at *Bioinformatics* online, were compared as the golden standard. From the experimental results, we concluded that the three innovations proposed in our study are effective (as detailed in the results section) and that the overall performance of our model is better than those of the previous optimal models (Chatzianastasis *et al.* 2023).

To prove the effectiveness of the hypergraph neural network and the use of biological pathways, we proposed a DGHNN model and conduct related experiments (Fig. 2), the results of which not only demonstrate the improved performance of the model after encoding and integrating biological pathway data but also prove the effectiveness of using hypergraph convolutional neural networks for DGHNN development.

To prove the effectiveness of the deep graph neural network, we first explored the effect of the number of layers on deep graph/hypergraph neural networks by conducting experiments and comparing the performance and time required for the DGHNN under different numbers of layers. The results show that a 27-layer deep graph and hypergraph convolutional neural network is the best configuration for pan-cancer related genes (Table 3, available as supplementary data at *Bioinformatics* online and Fig. 3). After that, we not only demonstrated that deep graph neural networks can more effectively obtain higher-order information from graphs and achieve better performance (Fig. 4) but also proved that our proposed skip residual connections can effectively alleviate the vanishing gradient problem that may occur in the training of deep graph neural networks (Fig. 5).

To prove the effectiveness of the feature tokenizer and transformer for classification, Fig. 6 shows comparisons between the DGHNN and DGHNN_L. Notably, the use of feature tokenizer and transformer for classification not only can improve the performance of the model but also has good compatibility for possible subsequent optimizations. Thus, if we can locate features that might be relevant to the prediction of pan-cancer related genes later, even if they are not related to the graph structure, we can still easily embed them into the overall prediction network as long as they can be recorded as tabular data (Gorishniy *et al.* 2021).

Although this study has made good progress, several shortcomings remain. For example, this study considered biological pathway network information and used hypergraphs to model and encode it, but the hidden information that may exist inside each biological pathway was ignored. In addition, owing to limited computing resources, the dimensions of feature embedding in the classifier are limited, which makes exploring the effect of using more dimensions for embedding impossible. Finally, several novel methods, such as HyperConnections (Zhu *et al.* 2025), may benefit the model used in this study, which is worth investigating in our future studies.

In summary, we propose a pan-cancer related gene predictive model based on a deep graph and hypergraph convolutional neural network, DGHNN, which not only has made good progress but also provides potential new methods and ideas for subsequent pan-cancer related gene predictive studies and other studies related to graph convolutional neural networks.

## Supplementary Material

btaf379_Supplementary_Data

## Data Availability

The data and code used in this study can be found in with DOI: 10.5281/zenodo.15533557 or accessed from https://github.com/skytea/DGHNN. The data are originated from https://owww.molgen.mpg.de/∼sasse/EMOGI/.
