# Supplementary material for: DGHNN: a deep graph and hypergraph neural network for pan-cancer related gene prediction
Source: Bioinformatics. 2025 Jun 28;41(7):btaf379. doi: 10.1093/bioinformatics/btaf379 (PMC12254129; doi:10.1093/bioinformatics/btaf379)
Supplement: btaf379_Supplementary_Data [file btaf379_supplementary_data.docx]

**Supplementary Material**

**SUPPLEMENTARY INFORMATION**

**Supplementary Methods**

**Method S1.** Graph and hypergraph neural network

**Method S2.** Deep graph and hypergraph neural network based on skip residual connection

**Method S3.** Classification module based on feature tokenizer and Transformer

**Method S4.** Datasets

**Supplementary Tables**

**Table S1.** AUPRC results of ablation experiment for all six datasets

**Table S2.** AUROC results of ablation experiment for all six datasets

**Table S3.** AUPRC results and time costs of different layers

**Supplementary Methods**

**Method S1. Graph and hypergraph neural network**

In a graph neural network, the features of each node are updated by aggregating the features of its neighbors. Suppose we have a graph $G=(V,E)$, where $V=\{v_{1},v_{2}\ldots\ldots,v_{n}\}$ is a set of nodes and $E$ is a set of edges. For each node $v_{i}$, the GNN update as Eq.1.

$$\begin{aligned} X^{\left( l+1 \right)}=\sigma\left( D^{-\frac{1}{2}}AD^{-\frac{1}{2}}X^{\left( l \right)}P^{\left( l \right)} \right) \end{aligned}(1)$$

$X^{(l)}$ and $X^{(l+1)}$ are the node eigenmatrices of layer $l$ and layer $l+1$; $D$ represents the degree matrix of the graph; $A$ represents the adjacency matrix of the graph and $P^{\left( l \right)}$represents the weight parameter matrix of layer $l$.

When modeling biological pathways, a biological pathway often consists of multiple interrelated genes, which makes it difficult for simple graph models to comprehensively interpret a biological pathway and embed multiple biological pathways into a graph, but hypergraph and hyperedge can characterize such data structure (Feng, et al., 2019).

Similar to an ordinary graph, a hypergraph $HG=(V,HE)$ has a set of nodes $V=\{v_{1},v_{2}\ldots\ldots,v_{n}\}$ and a set of hyperedges $HE=\left\{ {he}_{1},{he}_{2}\ldots\ldots,{he}_{m} \right\}.$The hypergraph is represented as a combination of these nodes and edges. In an ordinary graph, an edge can only connect two nodes, while an edge $e_{i}$ of a hypergraph can connect multiple nodes. And each hyperedge is represented as a set of nodes, indicating it can connect multiple nodes in the set.

$$\begin{aligned} he_{i}=\left\{ v_{1},v_{2}\ldots\ldots,v_{k} \right\} \end{aligned}(2)$$

As shown in equation 6, it represents a hyperedge $he_{i}$, which connects k nodes.

After defining hypergraphs, like ordinary graph convolutional neural networks, convolutional neural networks of hypergraphs can be defined as Eq.3.

$$\begin{aligned} X^{(l+1)}=\sigma(D_{v}^{-\frac{1}{2}}HWD_{e}^{-1}H^{T}D_{v}^{-\frac{1}{2}}X^{(l)}P^{\left( l \right)}) \end{aligned}(3)$$

$X^{(l)}$ and $X^{(l+1)}$ are the node eigenmatrices of layer $l$ and layer $l+1$; $H$ represents the incidence matrix of the hypergraph; $D_{v}$ represents the point degree matrix of hypergraph; $D_{e}$ represents the edge degree matrix of hypergraph and $P^{\left( l \right)}$ represents the weight parameter matrix of layer $l$.

**Method S2. Deep graph and hypergraph neural network based on skip residual connection**

For complex graph structures, GNN can learn the representation of nodes through the feature aggregation of neighboring nodes, but shallow neural networks are limited to the expression ability and cannot fully explore the relationship between higher-order nodes. Although deep graph neural networks can obtain more abundant graph information through multi-layer structure, with the increase of the number of layers, the problem of vanishing gradient will worsen, which makes it difficult to train the node features, and affects the performance of the model. Inspired by the idea of ResNet, we propose a deep graph neural network model based on skip residual connections (He, et al., 2015) to solve this problem, because not only vanishing gradient in deep networks can be effectively alleviated by skip residual connection, but also representation ability of the model on graph-structured data can be increased.

The structure of the skip residual connection is shown in Figure 1B, which consists of several graph or hypergraph convolution modules. Each module consists of two parts: one is the graph or hypergraph convolution network, which is used to capture complex relationships between nodes; The other part is based on linear transformation or identity residual connection, which is used to guarantee the smooth transmission of features.

When input and output features have the same dimension, we directly connect the original input as the residual to the feature vector after multiple graph or hypergraph convolution, which can be demonstrated as Eq.4

$$\begin{aligned} y=F_{Conv}\left( x, \left\{ W_{Conv} \right\} \right)+x \end{aligned}(4)$$

When the dimensions of the input and output features are different, linear transformation is used to map the original input to the corresponding dimension as a residual, and then connected to the feature vector after multiple graph or hypergraph convolution as Eq.5

$$\begin{aligned} y=F_{Conv}\left( x, \left\{ W_{Conv} \right\} \right)+W_{Linear}x \end{aligned}(5)$$

Here, $x$ represents the input of the module and $y$ represents the output of the module. The $F_{Conv}$ function represents the graph or hypergraph convolution, $W_{Conv}$ represents the parameters to be learned, and $W_{Linear}$ represents the parameters of linear transformation.

**Method S3. Classification module based on feature tokenizer and Transformer**

To build a predictive classifier for the numerical data, FT-transformer (Feature Tokenizer and Transformer) method proposed by Gorishniy et al (Gorishniy, et al., 2021) provides a potential method. FT-transformer can perform tokenization like LLMs, but carries out specific design for numerical data and tabular data, which enables these data to express features lossless in a suitable way (Gorishniy, et al., 2021). In addition, compared with the normal linear classifier (Alain and Bengio, 2016), feature tokenizer and transformer method can maintain good compatibility for possible new features which may be discovered later.

The key of the classification module is to tokenize the input features, which means each input feature will be transformed into a separate embedding, so that each feature can participate in the calculation process of the Transformer. For numerical features, we firstly use linear transformation to perform the embedding. And then, we concatenate all embedding vectors and the classification tag $E_{CLS}$, which is used for classification. After that, we input those embeddings into the Transformer. After calculation in the Transformer, the classification tag $E_{CLS}$ is employed to predict the classification.

For an n-dimensional numerical feature $X=\{x_{1},x_{2}\ldots\ldots,x_{n}\}$, the linear transformation of the numerical feature $x_{i}$ can be described as Eq.6

$$\begin{aligned} e_{i}=W_{embed}x_{i}+ b_{embed} \end{aligned}(6)$$

Here, $e_{i}$ represents the embedding of $x_{i}$, $W_{embed}$ and $b_{embed}$ are weight matrix and bias for feature embeddings to be learned, respectively.

Then, classification tag $E_{CLS}$ will be added into the embeddings and input embeddings into Transformer for calculation to obtain the fused feature $E_{embed}$. Finally, we extract the classification tag $E_{CLS}$ from $E_{embed}$ and predict final classification by Eq.7 and 8.

$$\begin{aligned} E_{embed}=Transformer(concat\left( E_{CLS}, Embed\left( X \right) \right)) \end{aligned}(7)$$

$$\begin{aligned} Pred=W_{cls}E_{CLS}+ b_{cls} \end{aligned}(8)$$

Here, $E_{CLS}$ represents the embedding of classification tag, $Pred$ represents the final prediction result, $W_{cls}$ and $b_{cls}$ are weight matrix and bias for classification to be learned, respectively.

**Method S4. Datasets**

To have a fair comparison with the previous methods (Chatzianastasis, et al., 2023; Schulte-Sasse, et al., 2021), we used the same data sets and the same data splits as theirs. Specifically, six datasets corresponding to six PPI networks are: CPDB (Kamburov, et al., 2011), Multinet (Khurana, et al., 2013), PCNet (Huang, et al., 2018), STRING-db (Szklarczyk, et al., 2019), Iref (Razick, et al., 2008) and its new version Iref (2015). The features of the gene nodes contain data on gene mutation frequency, copy number aberration (CNA), DNA methylation, and gene expression (GE) in 29,446 samples from 16 different cancer types. Positive examples of genes include cancer genes collated by experts, high-confidence cancer genes mined from PubMed abstracts, and genes with altered expression and promoter methylation in at least one cancer type. Negative examples of genes are obtained by removing genes which are not associated with cancer from a set of genes, and genes present in positive samples and various cancer databases are removed. For more information on data collection and processing methods, refer to the study by Schulte-Sasse et al (Schulte-Sasse, et al., 2021).

In addition, since the EMGNN model uses multiple graphs as inputs, when tested on each data set, the test nodes corresponding to the PPI network are remained as the testing set. The remaining nodes and nodes in other PPI networks are combined. Next, 90% of them are assigned to the training set and 10% to the validation set. We also used the same data processing method, which are detailed by Chatzianastasis et al (Chatzianastasis, et al., 2023).

**Supplementary Tables**

**Table S1**

Table S1. AUPRC results of ablation experiment for all six datasets

| Method | CPDB | Multinet | PCNet | STRING-db | Iref | Iref(2015) |
| --- | --- | --- | --- | --- | --- | --- |
| DGHNN | **0.828±0.009** | **0.873±0.003** | **0.780±0.007** | **0.859±0.006** | **0.823±0.009** | 0.826±0.004 |
| DGHNN_L | 0.817±0.008 | 0.857±0.006* | 0.764±0.013 | 0.848±0.006* | 0.813±0.007 | **0.829±0.003** |
| SGHNN | 0.805±0.008* | 0.865±0.003* | 0.778±0.009 | 0.841±0.007* | 0.814±0.006 | 0.822±0.005 |
| DGHNN_NO_H | 0.811±0.009* | 0.852±0.011* | 0.760±0.011* | 0.849±0.011 | 0.799±0.009* | 0.808±0.010* |
| DGHNN_NO_G | 0.569±0.110* | 0.600±0.157* | 0.467±0.060* | 0.539±0.074* | 0.515±0.072* | 0.638±0.045* |

The result with * represents the result of this method is different from the one of DGHNN on this dataset in statistical significance, which is calculated by T test and the threshold is set to 0.05.

**Table S2**

Table S2. AUROC results of ablation experiment for all six datasets

| Method | CPDB | Multinet | PCNet | STRING-db | Iref | Iref(2015) |
| --- | --- | --- | --- | --- | --- | --- |
| DGHNN | **0.924±0.004** | **0.962±0.003** | **0.949±0.001** | **0.937±0.003** | **0.944±0.006** | **0.925±0.002** |
| DGHNN_L | 0.919±0.004 | 0.959±0.001 | 0.945±0.002* | 0.930±0.004* | 0.943±0.002 | 0.925±0.002 |
| SGHNN | 0.920±0.003 | 0.961±0.000 | 0.948±0.001 | 0.927±0.003* | 0.946±0.002 | 0.921±0.003 |
| DGHNN_NO_H | 0.916±0.004* | 0.957±0.002* | 0.947±0.003 | 0.929±0.004* | 0.937±0.004* | 0.916±0.005* |
| DGHNN_NO_G | 0.806±0.062* | 0.894±0.055* | 0.874±0.021* | 0.804±0.047* | 0.862±0.029* | 0.840±0.028* |

The result with * represents the result of this method is different from the one of DGHNN on this dataset in statistical significance, which is calculated by T test and the threshold is set to 0.05.

**Table S3**

Table S3. AUPRC results and time costs of different layers

| Layers | AUPRC | Average computing Time (s) |
| --- | --- | --- |
| 1*3 | 0.814±0.006 | 6574 |
| 3*3 | 0.820±0.005 | 12486 |
| 6*3 | 0.820±0.006 | 17272 |
| **9*3** | **0.823±0.009** | **21005** |
| 12*3 | 0.812±0.007 | 30529 |

**Reference**

Alain, G. and Bengio, Y.J.A. Understanding intermediate layers using linear classifier probes. 2016;abs/1610.01644.

Chatzianastasis, M., Vazirgiannis, M. and Zhang, Z. Explainable Multilayer Graph Neural Network for cancer gene prediction. *Bioinformatics* 2023;39(11):btad643.

Feng, Y.*, et al.* Hypergraph Neural Networks. *Proceedings of the AAAI Conference on Artificial Intelligence* 2019;33(01):3558-3565.

Gorishniy, Y.*, et al.* Revisiting deep learning models for tabular data. *Nips '21* 2021.

He, K.*, et al.* Deep Residual Learning for Image Recognition. 2015:770-778.

Huang, J.K.*, et al.* Systematic Evaluation of Molecular Networks for Discovery of Disease Genes. *Cell systems* 2018;6(4):484-495.e485.

Kamburov, A.*, et al.* ConsensusPathDB: toward a more complete picture of cell biology. *Nucleic Acids Res* 2011;39(Database issue):D712-717.

Khurana, E.*, et al.* Interpretation of genomic variants using a unified biological network approach. *PLoS computational biology* 2013;9(3):e1002886.

Razick, S., Magklaras, G. and Donaldson, I.M. iRefIndex: a consolidated protein interaction database with provenance. *BMC Bioinformatics* 2008;9:405.

Schulte-Sasse, R.*, et al.* Integration of multiomics data with graph convolutional networks to identify new cancer genes and their associated molecular mechanisms. *Nature Machine Intelligence* 2021;3(6):513-526.

Szklarczyk, D.*, et al.* STRING v11: protein–protein association networks with increased coverage, supporting functional discovery in genome-wide experimental datasets. *Nucleic Acids Research* 2019;47(D1):D607-D613.
